# Evaluation of Sarcopenia by Psoas Muscle Measurements in Bullous Pemphigoid: A Single-Institution Survey in Japan

**DOI:** 10.7759/cureus.72452

**Published:** 2024-10-26

**Authors:** Yu Matsui, Teruhiko Makino, Tadamichi Shimizu

**Affiliations:** 1 Department of Dermatology, University of Toyama, Toyama, JPN

**Keywords:** bullous pemphigoid (bp), frailty syndrome, psoas muscle index, retrospective research, sarcopenia

## Abstract

Bullous pemphigoid (BP) is a rare autoimmune blistering disease that primarily affects elderly individuals. Based on the Bullous Pemphigoid Disease Area Index (BPDAI) severity assessment, immunosuppressive drugs are recommended for severe cases that fall within the more than moderate classes. Sarcopenia, which is characterized by decreased skeletal muscle mass and function in elderly patients, is a progressive and widespread skeletal muscle disease. We retrospectively surveyed patients who received treatment for BP at our hospital between 2012 and 2024 to evaluate the relevance of sarcopenia, as assessed using the psoas muscle mass index (PMI), and to investigate the benefit of relapse-free survival (RFS). A total of 59 patients with BP were included in this study. A total of 57.6% (34 of 59 patients) of the BP patients had sarcopenia, as measured by PMI. Kaplan-Meier analysis showed that the sarcopenia group (n = 34) had a median RFS of 394 days, which was not longer than that of the non-sarcopenia group (n = 25, 275 days) (p = 0.894). No significant difference in RFS was observed in the subgroup analysis based on the severity of BPDAI. Our real-world data confirmed the high prevalence of sarcopenia in the BP population and the efficacy of standard treatment centered on immunosuppressive therapy based on the severity of BPDAI. There is little basis for reducing oral corticosteroids below 0.5 mg/kg based solely on the patient’s perceived frailty, regardless of the severity of the BPDAI. Limited evidence is available to assess the association between sarcopenia and autoimmune diseases in terms of their etiology. As populations continue to age globally, it is important for clinicians to prioritize addressing diseases while considering whether patients experience frailty in daily practice.

## Introduction

Bullous pemphigoid (BP) is an autoimmune blistering disease that affects elderly patients, particularly those aged >70 years of age [1]. Based on the severity judgment by the Bullous Pemphigoid Disease Area Index (BPDAI), internal medicines, including immunosuppressive drugs (e.g., oral corticosteroids), are indicated for severe cases more frequently than moderate classes [2]. Clinicians may be hesitant to prescribe high doses of corticosteroids, which can prolong the tapering period. Side effects include continued susceptibility to various infections, skeletal muscle catabolism, and corticosteroid-induced osteoporosis [3]. Sarcopenia is a progressive and widespread skeletal muscle disease characterized by decreased skeletal muscle mass and function in elderly patients [4]. The psoas muscle mass index (PMI) is a relatively simple method of expressing total body skeletal muscle mass and is often used to quantify sarcopenia [5]. This index has been shown to predict the survival of cancer patients or postoperative patients. However, limited data are available for evaluating PMI in autoimmune blistering diseases. Recently, sarcopenia has been associated with several autoimmune diseases including rheumatoid arthritis (RA), inflammatory bowel disease (IBD), and type 1 diabetes (T1D) [6]. Therefore, we conducted a retrospective survey on the prognosis of BP patients and investigated its relevance to sarcopenia using the PMI.

## Materials and methods

Study population

Data were retrospectively collected from patients with BP hospitalized for treatment at Toyama University Hospital between April 2012 and May 2024. Patients treated only with topical steroids, those with mild BPDAI scores, and those who refused computerized tomography (CT) scans were excluded. The collected baseline characteristics included age, sex, body mass index (BMI), BPDAI, associated medical conditions (prescription history of dipeptidyl peptidase-4 inhibitor (DPP-4 inhibitor), and past medical history of neurological disorders), blood test results (anti-BP180 antibody titer, eosinophils, hemoglobin, and albumin), primary dose of oral corticosteroids, duration of hospitalization, and PMI. DPP-4 inhibitors linked to BP risk [7], anti-BP180 antibody titer, eosinophils, hemoglobin, albumin, and the presence of neurological complications are conceivable risk factors for recurrence [8,9]. The primary endpoint was relapse-free survival (RFS) from the first medical intervention to the date of recurrence, as stated by the Japanese Dermatological Association guidelines for BP or the last follow-up visit.

Assessment of the PMI

CT images were obtained for all subjects to exclude BP caused by malignant complications, the total psoas muscle cross-sectional area at the L3 level was measured, and PMI (mm2/m2) was calculated by normalizing muscle area (mm2) by height squared. Sarcopenia was defined as PMI < 6.36 cm2/m2 for males and 3.92 cm2/m2 in females, based on healthy young Asian adult standards [10].

Statistical analysis

Continuous variables were reported as medians and ranges, and group comparisons were performed using one-way ANOVA and χ2 tests. RFS was calculated using the Kaplan-Meier method, and log-rank tests were used to compare categorical variables. Statistical significance was set at P < 0.05. were considered statistically significant. All statistical analyses were performed with EZR version 1.53 [11] (Saitama Medical Center, Jichi Medical University, Saitama, Japan), which is a graphical user interface for R (The R Foundation for Statistical Computing, Vienna, Austria).

Ethical review

The Toyama University Hospital Ethics Board approved the study and waived patient consent in line with the Declaration of Helsinki (study number: R2024047; IRB approval date: June 17, 2024).

## Results

Patient characteristics

Table 1 details clinical data from 59 patients, divided into sarcopenia (34, 57.6%) and non-sarcopenia (25, 42.4%) groups. Patients were all Japanese (33 men {55.9%} and 26 women {44.1%}); the median age was 77.0 years (range: 47-93).

**Table 1:** Patient demographics and baseline characteristics. PMI, psoas muscle mass index; BPDAI, Bullous Pemphigoid Disease Area Index; DPP-4, dipeptidyl peptidase-4; PSL, prednisolone. * Chi-square test; for tests other than the Chi-squared test, One-Way Analysis of Variance (One-Way ANOVA) was used.

Patient characteristics		Patients (n = 59)	Sarcopenia group	Non-sarcopenia group	P value
						Patients (n = 59)		59	34 (57.6)	25 (42.4)	
Sex					0.06*
	Male (%)	33 (55.9)	23 (67.6)	10 (40.0)	
	Female (%)	26 (44.1)	11 (32.4)	15 (60.0)	
Age	Median (range)	77 (47–93)	79 (60–93)	76 (47–92)	0.116
BMI	Median (range)	23.3 (14.8–31.5)	22.5 (14.8–31.5)	23.7 (18.9–29.6)	0.18
PMI	Male median (range)	5.84 (1.60-10.95)	5.45 (1.60-6.23)	8.41 (5.43-10.95)	<0.01
	Female median (range)	4.19 (2.05-8.32)	3.24 (2.05-3.86)	5.09 (4.00-8.32)	<0.01
BPDAI	Moderate (%)	27 (45.8)	16 (47.1)	11 (44.0)	1*
	Severe (%)	32 (54.2)	18 (52.9)	14 (56.0)	
Blood test	Anti-bp180 antibody (range)	372 (9-31300)	445.7 (9-31300)	159.4 (19-29700)	0.696
	Eosinophil count (range)	810 (0-13300)	785 (0-13300)	890 (0-10960)	0.787
	Hemoglobin (range)	13.1 (7.6-18.5)	13.0 (7.6-18.5)	13.1 (9.6-17.7)	0.597
	Albumin (range)	3.4 (1.2-4.6)	3.3 (2.0-4.6)	3.7 (1.2-4.5)	0.297
Complication	Neurological disease (%)	15 (25.4)	9 (26.4)	6 (24.0)	
Internal medicine	DPP4-inhibitors (%)	19 (32.2)	10 (29.4)	9 (36.0)	
Treatment	Initial dosage of PSL (mg/kg)	0.53 (0.16-1.79)	0.55 (0.16-1.79)	0.52(0.16-0.68)	0.05
	median hospitalization (range)	50.7 (10–723)	59.8 (15-723)	38.5 (10-149)	0.397

The severity was moderate in 27 (45.8%) and severe in 32 (54.2%) patients. PMI was significantly lower in the sarcopenia group in both sexes (males: 5.45 {1.60-6.23} vs. 8.41 {5.43-10.95}, p<0.001; females: 3.23 {range 2.05-3.86} vs. 5.09 {4.00-8.32}, p<0.001). No significant differences were found in age, BMI, BPDAI severity, blood test results, associated medical conditions, primary dose of oral corticosteroids, or hospitalization duration.

Survival data

**Figure 1:**
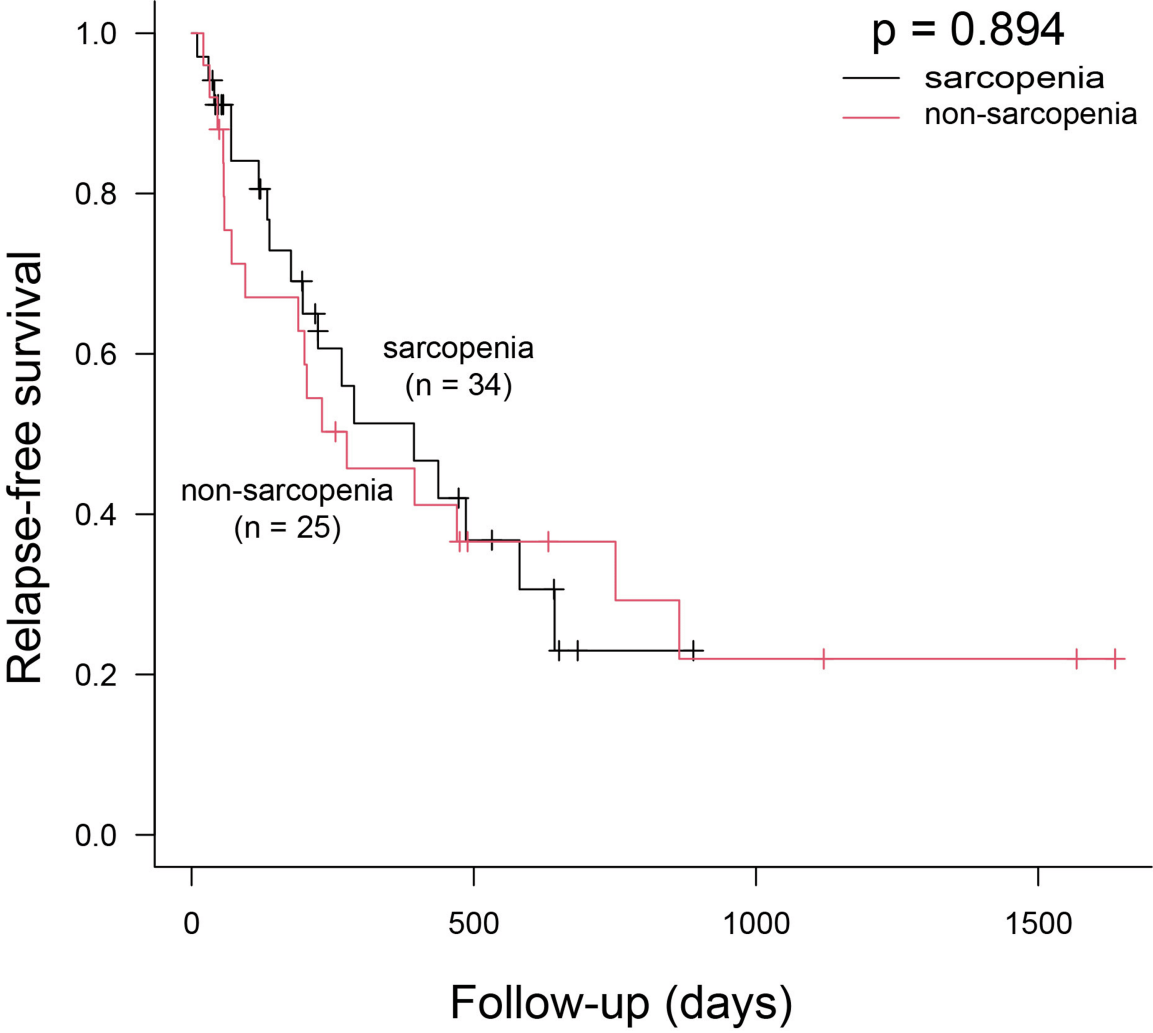
Relapse-free survival (RFS). From the date of the first medical intervention to the date of recurrence of bullous pemphigoid (BP) or the last follow-up visit. Patients were divided into the sarcopenia group and the non-sarcopenia group. Median RFS was 394 days (95% CI, 176–643) for the sarcopenia group and 275 days (95% CI, 71–751) for the non-sarcopenia group.

**Figure 2:**
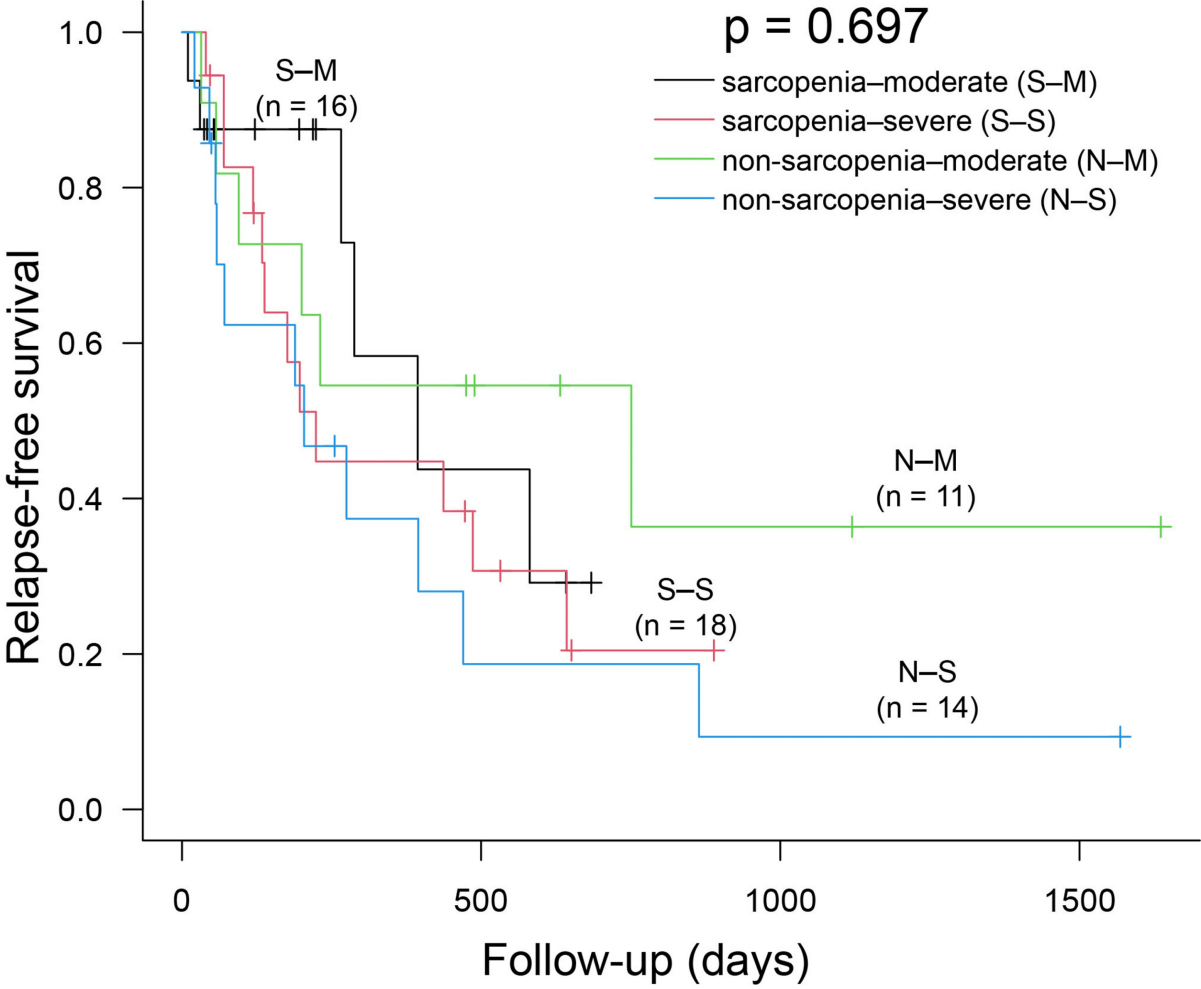
Subgroup analysis based on Bullous Pemphigoid Disease Area Index severity. Relapse-free survival (RFS) in patients according to the severity of the Bullous Pemphigoid Disease Area Index. Patients were divided into the four following groups: sarcopenia–moderate {S–M group}, n = 16; sarcopenia–severe {S–S group}, n = 18; non-sarcopenia–moderate {N–M group} n = 11; and non-sarcopenia-severe {N–S group}, n = 14. The median RFS was 394 days (95% CI, 266–not available {NA}), 224 days (95% CI, 119–643), 751 days (95% CI, 57–NA), and 204 days (95% CI, 56–470).

A Kaplan-Meier analysis showed no significant differences in RFS between the groups (p = 0.894) (Figure 1). The median RFS was 394 days (95% CI, 176-643) in the sarcopenia group and 275 days (95% CI, 71-751) in the non-sarcopenia group. A subgroup analysis based on BPDAI severity also showed no significant differences (p = 0.697) (Figure 2).

## Discussion

Our analysis suggests two new perspectives by highlighting the relationship between sarcopenia measured by the PMI and BP. First, there is a high prevalence of sarcopenia in the BP population and its association with the etiology. Sarcopenia, defined as age-related changes in the skeletal muscle, is an important disease concept in geriatrics and aging medicine. However, in the field of dermatology, few clinical studies have examined sarcopenia in relation to autoimmune diseases. Based on 263 systematic reviews and meta-analyses conducted worldwide, primarily in Europe and Asia, the prevalence of sarcopenia has been reported to be 10-27% in individuals ≥ 60 years of age [12]. In Japan, a study based on a group health examination involving 1,851 individuals reported the prevalence of sarcopenia to be 11.5% (105/917) in men and 16.7% (156/934) in women [13]. In comparison to previous studies, the complication rate of sarcopenia in patients with BP is likely to be higher than that in the normal population. Although BP is mainly affected by elderly age, 57.6% (34 of 59 patients) of BP patients had sarcopenia, as measured by the PMI. The report by Hamaguchi et al., which we also used as a reference for diagnosing sarcopenia based on the PMI, is frequently cited in Japan and other regions of Asia [10]. This study proposed cutoff values for sarcopenia based on an analysis of 541 living liver transplant donors. Since the study focused on healthy individuals eligible to donate liver tissue, it is possible that the cutoff values are higher than the general population, suggesting that the prevalence of sarcopenia in BP patients may be even higher.

The causal relationship between aging and various biological changes remains ambiguous; however, aging is characterized by a decline in the function of multiple systems, organs, and tissues throughout the body, which is associated with reduced adaptability and diminished innate immunity [14]. Dysfunction in skeletal muscle tissue may trigger the onset of a chronic inflammatory response, which, in turn, further impairs skeletal muscle function, creating a self-perpetuating cycle. Accumulating evidence suggests that the low-grade chronic inflammation associated with aging may play a central role in the pathogenesis of sarcopenia [15]. The phenomenon of tissue injury secondary to a primary inflammatory process, which leads to the exposure of previously sequestered antigens and subsequently triggers a secondary autoimmune response, is known as "epitope spreading". This mechanism is considered to be a contributing factor in the pathogenesis of BP [16]. At the core of this theory is the well-known fact that in the skin of elderly individuals, the deterioration of epidermal cell-cell junctions contributes to greater skin fragility [17]. Even with age-related changes alone, there is an increase in low-grade chronic inflammation [18,19]. Additionally, it is hypothesized that inflammation may expose antigens, potentially promoting the production of BP180NC16a antibodies and other related antibodies, which are responsible for the development of BP [16,20]. A previous two-sample Mendelian randomization study implicated sarcopenia as a cause of autoimmune diseases, including RA, IBD, and T1D [6]. Although the etiology of sarcopenia in autoimmune diseases is unclear, autoimmune diseases with persistent chronic inflammation have been suggested as risk factors for sarcopenia [21]. There are scattered reports of increased blood levels of inflammatory cytokines (e.g., TNF-α and IL-6) in patients with sarcopenia in comparison to those without sarcopenia, suggesting a close relationship between sarcopenia and autoimmune diseases [22,23]. Under physiological conditions, pro-inflammatory cytokines play a crucial role in maintaining the balance between anabolic and catabolic processes in skeletal muscle. However, during conditions of muscle wasting such as sarcopenia, the expression of these cytokines is upregulated, leading to an increase in catabolic activity. Additionally, pro-inflammatory cytokines can inhibit protein synthesis within skeletal muscle cells, compromising muscle integrity and function, and ultimately contributing to the development of sarcopenia [24]. BP is a disease that frequently occurs in the elderly. However, instead of relying solely on chronological age as a measure, incorporating the presence of sarcopenia into the assessment may lead to a more accurate and timely diagnosis.

The second perspective, in terms of RFS, is the recommendation of guideline-compliant standard treatment [2]. There is little basis for starting oral corticosteroids at a dose below 0.5 mg/kg based on a patient’s impression of frailty, regardless of the severity of the BPDAI. There were no significant differences in RFS between patients with and without sarcopenia or in the subgroup analysis. There was also no statistically significant difference in the initial dose of oral corticosteroids between the sarcopenic and non-sarcopenic groups. In some cases, due to concerns about side effects, treatment may be initiated at lower doses than those recommended by the guidelines for patients who appear frail. The lack of a difference in the RFS of BP patients regardless of the presence or absence of sarcopenia based on the PMI suggests that the administration of moderate to high doses of oral corticosteroids in accordance with the assessment of the severity of BPDAI is a valid treatment option. For primary physicians responsible for managing the numerous side effects brought on by high-dose oral corticosteroids, the frail impression they get from patients can easily serve as a convenient excuse for prescribing low-dose oral steroids, even when such treatment offers little benefit. We should be mindful of this and base our treatment decisions on objective indicators such as BPDAI and antibody titers. The present study has several limitations. This was not a prospective study, and PMI has been reported to be useful as a convenient surrogate index for sarcopenia in many cases, although it does not have the same definition as sarcopenia. Data collected on PMI were determined using manual tracing; however, the correlation between PMI and total skeletal muscle mass was not determined.

## Conclusions

We demonstrated that the prevalence of sarcopenia determined using PMI-based indices in patients with BP was as high as 57.6%. Despite the high percentage, there were no significant differences in the RFS rates between BP patients with and without sarcopenia, and the severity of BP was not found to be associated with RFS. Although sarcopenia is a frailty-related factor that physicians receive from patients, it is unlikely to be a prognostic indicator of recurrent BP. Appropriate therapeutic interventions based on disease severity, as suggested by the guidelines, are considered important.
